# Soluble factors from adipose tissue-derived mesenchymal stem cells promote canine hepatocellular carcinoma cell proliferation and invasion

**DOI:** 10.1371/journal.pone.0191539

**Published:** 2018-01-18

**Authors:** Takahiro Teshima, Hirotaka Matsumoto, Hidekazu Koyama

**Affiliations:** Laboratory of Veterinary Internal Medicine, Department of Veterinary Clinical Medicine, School of Veterinary Medicine, Faculty of Veterinary Science, Nippon Veterinary and Life Science University, Musashino-shi, Tokyo, Japan; University of Navarra School of Medicine and Center for Applied Medical Research (CIMA), SPAIN

## Abstract

The potential effects of adipose tissue-derived mesenchymal stem cells (AT-MSCs) on the growth and invasion of canine tumours including hepatocellular carcinoma (HCC) are not yet understood. Moreover in humans, the functional contribution of AT-MSCs to malignancies remains controversial. The purpose of this study was to investigate the effects of AT-MSCs on the proliferation and invasion of canine HCC cells *in vitro*. The effect of AT-MSCs on mRNA levels of factors related to HCC progression were also evaluated. Conditioned medium from AT-MSCs (AT-MSC-CM) significantly enhanced canine HCC cell proliferation and invasion. Moreover, mRNA expression levels of transforming growth factor-beta 1, epidermal growth factor A, hepatocyte growth factor, platelet-derived growth factor-beta, vascular endothelial growth factor, and insulin-like growth factor 2 were 2.3 ± 0.4, 2.0 ± 0.5, 5.7 ± 1.9, 1.7 ± 0.2, 2.1 ± 0.4, and 1.4 ± 0.3 times higher, respectively (*P* < 0.05). The mRNA expression level of MMP-2 also increased (to 4.0 ± 1.2 times control levels) in canine HCC cells co-cultured with AT-MSCs, but MMP-9 mRNA significantly decreased (to 0.5 ± 0.1 times control levels). These findings suggest that soluble factors from AT-MSCs promote the proliferation and invasion of canine HCC cells.

## Introduction

Hepatocellular carcinoma (HCC) is the most common primary liver tumour in dogs, accounting for 50% of cases [1]. The prognosis for dogs with massive HCC following liver lobectomy is good [1,2]. Conversely, the prognosis for dogs with nodular and diffuse HCC is poor, as surgical resection is usually not possible because of the involvement of multiple liver lobes. Therefore, more effective therapeutic strategies are required.

Mesenchymal stem cells can be isolated from adipose tissue in dogs, as in humans [3–5]. Recently, adipose tissue-derived mesenchymal stem cells (AT-MSCs) were reported to be a source of cells that can be used therapeutically for tissue regeneration [4,6]. Indeed, several reports have indicated that AT-MSCs can ameliorate liver injury and cirrhosis in humans and rodents [7–12]. However, the functional effects of AT-MSCs on tumour cells remain unclear. A few studies have examined the effects of AT-MSCs on HCC in humans; however, their findings are controversial [13–16]. Moreover, there have been no reports of the effects of canine AT-MSCs on canine HCC. This study examined the effects of conditioned medium from canine AT-MSCs on the growth and invasion of canine HCC cells, and on mRNA expression levels of factors related to tumour progression in HCC cells.

## Materials and methods

### Canine AT-MSC isolation and culture

All experimental protocols involving the use of dogs were approved by the Bioethics Committee at Nippon Veterinary and Life Science University. Six healthy beagles (three males and three females; mean age 1.5 years; mean body weight 9.2 kg) (ORIENTAL YEAST, Tokyo, Japan) were included in this study.

Adipose tissue was aseptically collected from the falciform ligament fat of the six anaesthetised dogs. The tissue was washed extensively with PBS, minced, and digested with collagenase type I (Sigma-Aldrich, St. Louis, MO) at 37°C for 45 min with intermittent shaking. After washing with PBS and centrifuging, the pellets, containing the stromal vascular fraction, were resuspended, filtered through 100-μm nylon mesh and incubated overnight in high glucose Dulbecco’s Modified Eagle’s medium (H-DMEM) supplemented with 10% foetal bovine serum (FBS; Nichirei Bioscience, Tokyo, Japan) and 1% antibiotic–antimycotic (Thermo Fisher Scientific, Waltham, MA) in a humidified atmosphere of 5% CO_2_ at 37°C. Unattached cells were removed by changing the medium, and the attached cells were washed twice with PBS. Thereafter, the medium was replaced every 3–4 days. When the cells reached 80%–90% confluence, they were detached with trypsin-EDTA solution (Sigma-Aldrich, St. Louis, MO) and passaged.

### Characterisation of surface markers of AT-MSCs

Passage 2 AT-MSCs were analysed by flow cytometry. The cells were placed in fluorescence-activated cell sorting (FACS) tubes (BD Biosciences, Franklin Lakes, NJ) (2 x 10^5^ cells/tube) and washed with FACS buffer (PBS containing 2% FBS), blocking Fc receptors with canine Fc receptor binding inhibitor (Thermo Fisher Scientific, Waltham, MA), and then incubated with the fluorescein (FITC)- or phycoerythrin (PE)-conjugated antibodies [17,18] or their respective isotype controls listed in Table 1. The cells were washed twice with FACS buffer and resuspended in 500 μl of FACS buffer. Cell fluorescence was evaluated by flow cytometry in a FACSCalibur instrument (BD Biosciences, Franklin Lakes, NJ). Data were analysed using WinMDI 2.9 analysis software.

**Table 1:** List of antibodies for cell surface markers used in the study.

Antibody	Clone	Isotype	Source
CD14-FITC	M5E2	Mouse IgG2a	BD Pharmingen
CD29-PE	TS2/16	Mouse IgG1	BioLegend
CD34-PE	1H6	Mouse IgG1	R&D Systems
CD44-PE	IM7	Rat IgG2b	BioLegend
CD45-FITC	YKIX716.13	Rat IgG2b	eBioscience
CD90-PE	YKIX337.217	Rat IgG2b	eBioscience

### Differentiation assay

For osteogenic differentiation, passage 2 AT-MSCs were seeded on 6-well plates (5.0 x 10^3^ cells/cm^2^) and incubated in H-DMEM supplemented with 10% FBS and 1% antibiotic–antimycotic solution for 24 h. The medium was then changed to osteogenic medium (Cell Applications, San Diego, CA) [18]. The medium was changed twice-weekly. For osteogenic analysis, mineral deposits were quantitatively analysed by von Kossa staining after 21 days.

For adipogenic differentiation, passage 2 AT-MSCs were seeded on 6-well plates (8 x 10^3^ cells/cm^2^). The cells were cultured in H-DMEM supplemented with 10% FBS and 1% antibiotic–antimycotic solution until they reached confluence, and then the medium was changed to canine adipocyte differentiation medium (Cell Applications, San Diego, CA) [18]. The medium was changed twice-weekly. Adipogenesis was analysed by Oil Red O staining after 21 days.

### AT-MSC-conditioned medium

To prepare the AT-MSC-conditioned medium (AT-MSC-CM), passage 2 of AT-MSCs from each of the six dogs were seeded (3.0 x 10^4^ cells/cm^2^) individually in H-DMEM supplemented with 10% FBS and 1% antibiotic–antimycotic solution and incubated overnight. Adherent AT-MSCs were washed and further incubated in FBS-free H-DMEM for 36 h, and then the medium was collected individually and filtered through a 0.45-μm filter. A total of six stocks of AT-MSC-CM from each of the beagle dogs were stored for experimental use.

### Tumour cells and culture conditions

AZACH (Cosmo Bio, Tokyo, Japan), a canine HCC cell line, was cultured in H-DMEM supplemented with 10% FBS and 1% antibiotic–antimycotic solution in a humidified atmosphere of 5% CO_2_ at 37°C. The cell line was established from the tissue of a 12-year-old female Maltese dog with hepatic masses diagnosed as solid HCC by histopathology [19].

### Proliferation assay

Cell proliferation was determined using an MTT assay kit (Roche Diagnostics, Tokyo, Japan). AZACH cells were seeded in 96-well flat-bottomed plates (5 x 10^3^ cells/well). AT-MSC-CM was added to the AZACH cell culture medium to a final concentration of 0%, 10%, 30%, or 50%. Every 24 h for 3 days, MTT assays were performed according to the manufacturer’s instructions.

### Cell invasion assay

Cell invasion was assayed using a Transwell system (Corning, Corning, NY). Cells (2.5 x 10^4^) were resuspended in 500 μl of FBS-free H-DMEM and seeded into the Matrigel-coated upper chamber and control insert chamber. H-DMEM (750 μl) containing 10% FBS and AT-MSC-CM (0% or 30%) was added to the lower chamber and cells were cultured for 24 h. After incubation, the cells remaining in the upper chamber or on the upper membrane of the inserts were carefully removed with cotton swabs. Cells that had invaded across the Matrigel and passed through the Transwell filter were fixed in methanol and stained with modified Giemsa using a Diff-Quik stain kit (Sysmex, Hyogo, Japan). Cells adhering to the lower membrane of the inserts were counted in five non-overlapping fields under a light microscope at 200x magnification. Data are expressed as the percent of cells that invaded through the Matrigel-coated insert membrane relative to the control cells that migrated through the control membrane.

### Real-time quantitative reverse transcription (RT)-PCR

Canine HCC cells were seeded in 6-well plates (5.0 x 10^5^ cells/well) and AT-MSC-CM was added to a final concentration of 0% or 30%. Cells were incubated for 48 h, and then total RNA was extracted using a TRIzol Plus RNA Purification kit (Thermo Fisher Scientific, Waltham, MA) according to the manufacturer’s instruction. cDNA was synthesized from 0.5μg total RNA using random primers and a GoScript Reverse Transcriptase system (Promega, Madison, WI) according the manufacturer’s instructions. Real-time RT-PCR analyses were performed using SYBR Green Real-time PCR Master Mix (Promega, Madison, WI) to determine the mRNA levels of transforming growth factor-beta 1 (TGFβ1), epidermal growth factor (EGF), hepatocyte growth factor (HGF), platelet-derived growth factor-beta (PDGFβ), vascular endothelial growth factor (VEGF) A, insulin-like growth factor (IGF) 2, matrix metalloproteinase (MMP) 2, and MMP9. The primer sequences are listed in Table 2. The amplification conditions were 95°C for 2 min, followed by 40 cycles of 95°C for 15 sec and 60°C for 60 sec. After 40 cycles, a dissociation curve was generated to verify the specificity of each primer. All reactions were performed in duplicate. Expression levels of target genes were normalised to the level of glyceraldehyde 3-phosphate dehydrogenase and quantified with the ΔΔCt method.

**Table 2:** Primers used for real-time quantitative RT-PCR.

Gene		Sequence (5’-3’)	Length (bp)	Accession number
TGFβ1	For	CCTGCTGAGGCTCAAGTTAAAAG	81	L34956
	Rev	CTGAGGTAGCGCCAGGAATC		
EGF	For	CTATGGCCCTCAAGGATGGTG	125	NM_001003094
	Rev	GCAGCCTTGCTCTGTGTCCTTA		
HGF	For	AAAGGAGATGAGAAACGCAAACAG	95	AB090353
	Rev	GGCCTAGCAAGCTTCAGTAATACC		
PDGFβ	For	TACGAGATGCTGAGCGACCAC	112	NM_001003383
	Rev	ATCGGGTCAAATTCAGGTCCAA		
VEGFA	For	TTGCTGCTCTACCTCCACCAT	64	NM_001003175
	Rev	TGTGCTCTCCTCCTGCCATAG		
IGF2	For	GGAAGAGTGCTGTTTCCGTA	125	XM_546602
	Rev	GGGTATCTGGGGAAGTTGTC		
MMP2	For	ACTTTGATGACGATGAGCTATGGA	73	XM_014109407
	Rev	CCGTCGGCATTCCCATACT		
MMP9	For	CTGGAGAGCTGGACAAAACC	232	NM_001003219
	Rev	TACACGCGAGTGAAGGTGAG		
GAPDH^a^	For	GATGGGCGTGAACCATGAG	131	NM_001003142
	Rev	TCATGAGGCCCTCCACGAT		
GUS^a^	For	CCTCCTGCCGTATTACCCTTG	117	NM_001003191
	Rev	TCTGGACGAAGTAACCCTTGG		
RPS5^a^	For	TCACTGGTGAGAACCCCCT	141	XM_533568
	Rev	CCTGATTCACACGGCGTAG		

^a^ Reference genes. Glyceraldehyde 3-phosphate dehydrogenase (GAPDH) was identified as the most stable reference gene for normalization of relative mRNA concentration using the basic GeNorm Visual application for Microsoft Excel.

### Statistical analysis

All data are presented as mean ± standard deviation. Differences between two groups were analysed with the Student’s t-test. Differences among multiple groups were analysed by one-way analysis of variance with a post-hoc Tukey–Kramer test. *P* < 0.05 was considered statistically significant. Statistical analyses were performed using Excel 2010 with Statcel 3 add-in software (OMS, Saitama, Japan). All data are representative of three independent experiments.

## Results

### Characterisation of AT-MSCs

**Fig 1:**
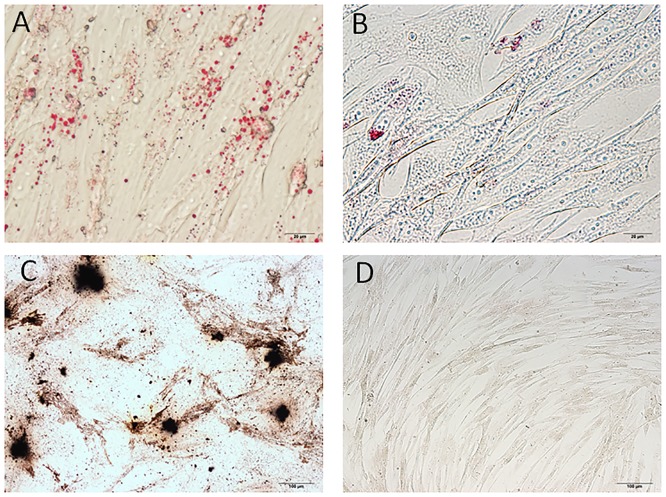
Multilineage differentiation of canine adipose tissue-derived mesenchymal stem cells (AT-MSCs). (A) Adipogenic differentiation was identified by Oil Red O staining. (B) Oil Red O staining of control undifferentiated cells. (C) Osteogenic differentiation was identified by von Kossa staining. (D) Von Kossa staining of control undifferentiated cells.

AT-MSCs from all six beagles were successfully cultured and expanded. The majority of the cells expressed the established mesenchymal stem cell markers CD29 (96.18 ± 1.03%), CD44 (99.48 ± 0.28%), and CD90 (94.03 ± 0.77%), and very few expressed CD14 (1.18 ± 0.07%), CD34 (0.71 ± 0.07%), or CD45 (1.02 ± 0.09%). The expression levels of cell markers in each AT-MSC line are shown in Table 3. The AT-MSCs exhibited multilineage plasticity, as demonstrated by their potential for adipogenic and osteogenic differentiation, compared with undifferentiated cells (Fig 1).

**Table 3:** Expression levels of cell surface markers in the six AT-MSC lines.

AT-MSC	CD29	CD44	CD90	CD14	CD34	CD45
1	96.5	99.7	94.6	1.1	0.8	1.1
2	97.2	99.4	93.1	1.2	0.7	1.1
3	94.2	98.9	93.8	1.3	0.7	0.9
4	95.6	99.7	94.2	1.2	0.6	1.1
5	97.1	99.6	95.3	1.1	0.8	1.0
6	96.5	99.6	93.2	1.2	0.7	0.9

The data are presented as % of positive cells compared with AT-MSCs stained by individual isotype controls.

### Effects of AT-MSC-CM on canine HCC cell proliferation

**Fig 2:**
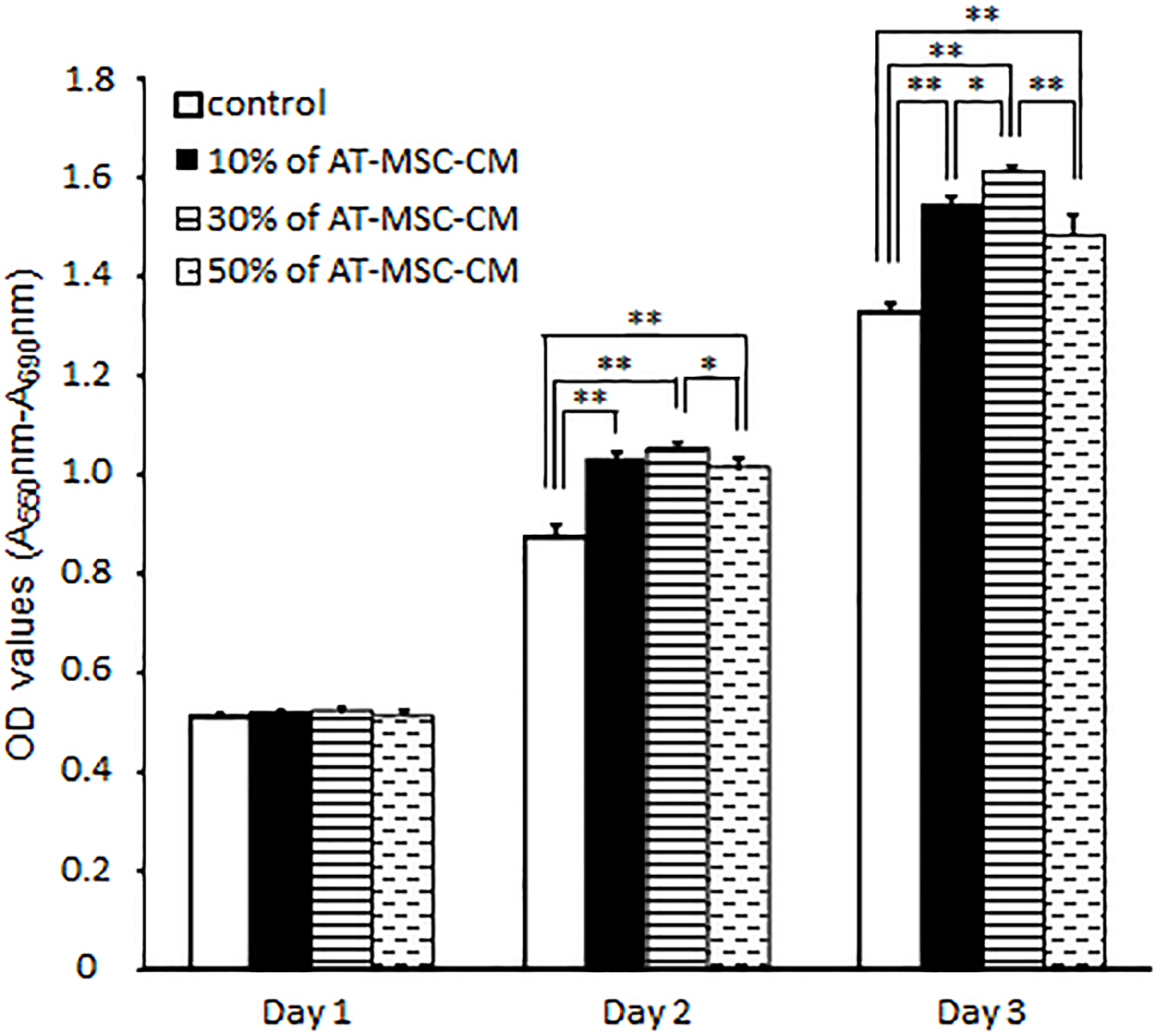
Effects of canine AT-MSC-CM on canine hepatocellular carcinoma cell proliferation. AZACH cells were cultured with various concentrations of AT-MSC-CM (10%, 30%, or 50% compared with 0% as control) for 72 h. Data are expressed as mean ± SD. *P < 0.05; **P < 0.01.

We next evaluated the effect of various concentrations of AT-MSC-CM (10%, 30%, or 50%) on the proliferation of AZACH cells, a canine HCC cell line, using MTT assays (Fig 2). On day 1, there were no significant differences among the culture conditions. However, on day 2 and 3, the OD values for the MTT assays in canine HCC cells cultured with AT-MSC-CM were significantly higher than cells cultured without AT-MSC-CM (*P* < 0.01). On day 3, the OD values for the cells cultured with 10%, 30%, and 50% AT-MSC-CM were 1.54 ± 0.02, 1.61 ± 0.01, and 1.48 ± 0.04, respectively, compared with 1.33 ± 0.02 in the cells cultured in the absence of AT-MSC-CM. Exposure of cells to 30% AT-MSC-CM resulted in the significantly increased the cell proliferation of the canine HCC cell line compared with cells cultured in the absence of AT-MSC-CM.

### Effects of AT-MSC-CM on canine HCC cell invasion ability

**Fig 3:**
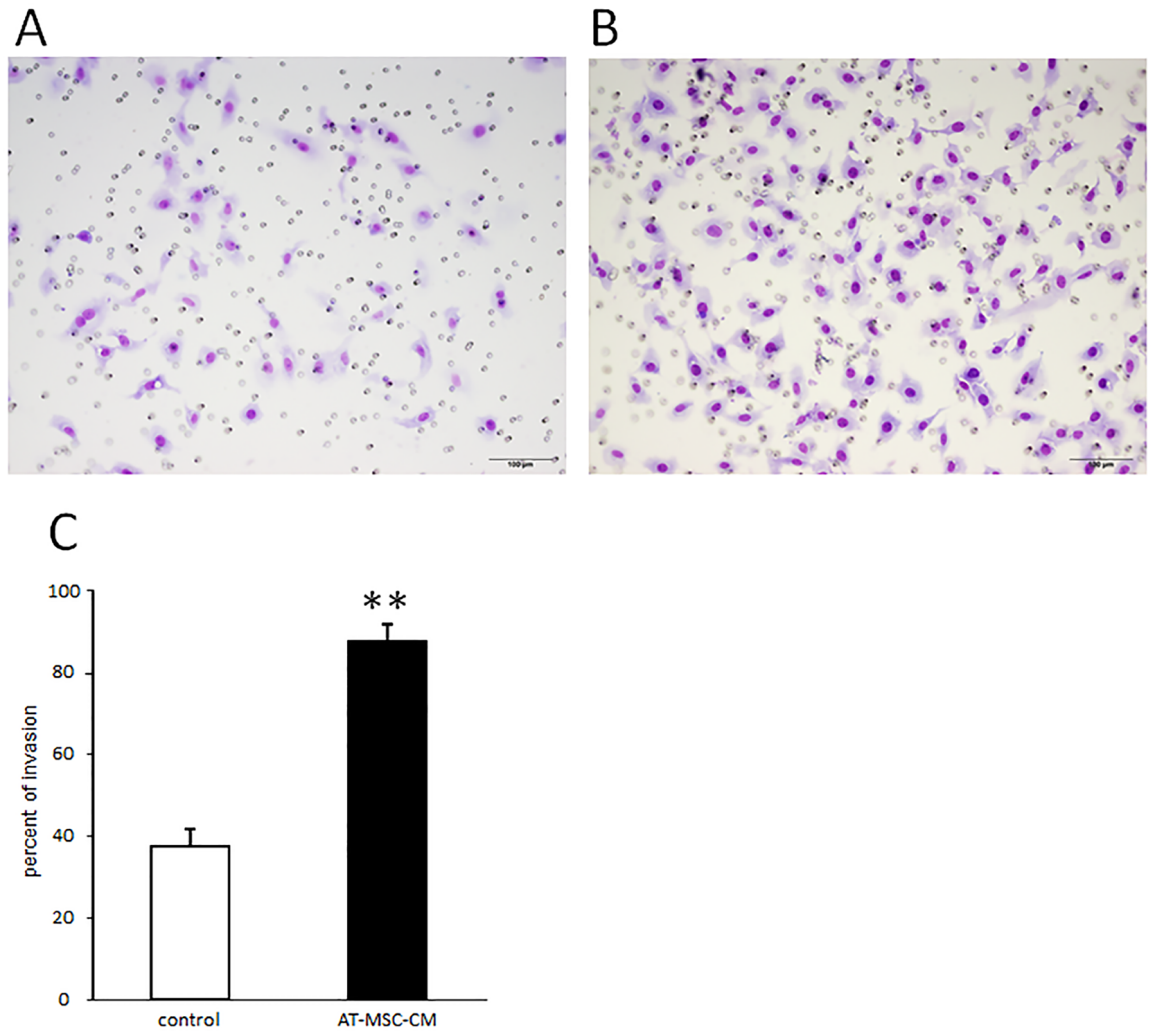
Effects of canine AT-MSC-CM on invasive ability of canine HCC cells. (A, B) Representative images of Transwell assays observed under a light microscope in the cultures without (A) and (B) with AT-MSC-CM. (C) The percent of invading HCC cells was significantly increased in cells cultured with AT-MSC-CM compared with control cells cultured without AT-MSC-CM. Data are expressed as mean ± SD. **P < 0.01.

Transwell invasion assay showed that the number of invading canine HCC cells was much higher for cells cultured with AT-MSC-CM than for cells cultured without AT-MSC-CM (Fig 3A and 3B). The percentage of invading cells cultured with 30% AT-MSC-CM (87.8 ± 5.3%) was significantly higher that of cells cultured without AT-MSC-CM (38.2 ± 4.3%) (*P* < 0.01; Fig 3C).

### Effects of AT-MSC-CM on growth factor mRNA expression in canine HCC cells

**Fig 4:**
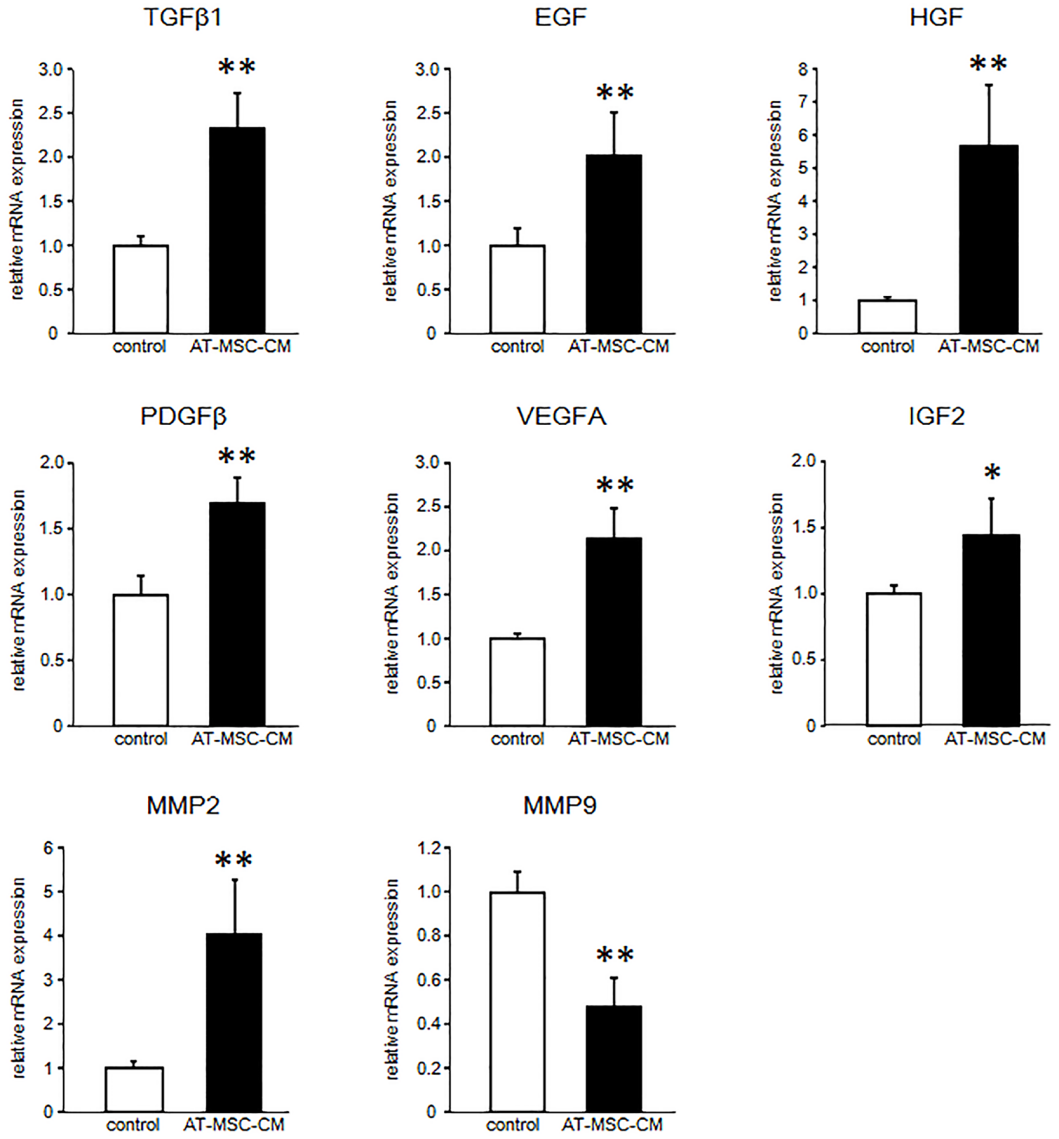
Growth factor and MMP mRNA expression in canine HCC cells cultured with AT-MSC-CM. Expression of mRNA of the indicated growth factors and MMPs were evaluated in canine HCC cells cultured with AT-MSC-CM compared with control HCC cells cultured without AT-MSC-CM. Data are expressed as mean ± SD. *P < 0.05; **P < 0.01.

After 48 h of culture, mRNA expression levels of growth factors were significantly increased in canine HCC cells cultured with 30% AT-MSC-CM (Fig 4). TGFβ1, EGF, HGF, PDGFβ, VEGFA, and IGF2 mRNA expression levels in the cells cultured with 30% AT-MSC-CM were 2.33 ± 0.41, 2.02 ± 0.49, 5.66 ± 1.87, 1.69 ± 0.20, 2.13 ± 0.35, and 1.39 ± 0.28 times higher, respectively, than those of cells cultured without AT-MSC-CM (*P* < 0.05).

### Effects of AT-MSC-CM on MMP2 and MMP9 mRNA expression in canine HCC cells

After 48 h of culture with 30% AT-MSC-CM, the expression level of MMP2 mRNA in canine HCC cells was increased (4.04 ± 1.23 times higher than the level in controls), but the expression levels of MMP9 mRNA was decreased compared with controls (0.48 ± 0.13 the level in cells cultured without AT-MSC-CM) (*P* < 0.05; Fig 4).

## Discussion

Our findings demonstrate that soluble factors from AT-MSCs efficiently promote canine HCC cell proliferation and invasion *in vitro*. AT-MSCs secrete various soluble factors including cytokines, chemokines, and growth factors [20,21]. However, whether the combination of soluble factors secreted by AT-MSCs would inhibit or promote tumour activity was unclear. Therefore, here we investigated the effects of AT-MSC paracrine activity on HCC cell proliferation and invasion using conditioned medium from AT-MSCs.

We initially studied the proliferation of HCC cells cultured with AT-MSC-CM. Over a 3 day period, AT-MSC-CM significantly enhanced cell proliferation, with 30% AT-MSC-CM producing the greatest enhancement. Therefore, 30% AT-MSC-CM was used for subsequent experiments. A Transwell cell invasion assay demonstrated that soluble factors from AT-MSCs also enhanced the invasiveness of HCC cells. Our cell proliferation and invasion assay results of canine HCC cells incubated with AT-MSC-CM differ from those of studies using human HCC cell lines [13,16].

In a study using five human HCC cell lines, proliferation of all cell lines was inhibited by AT-MSC-CM [16]. Another study of one human HCC cell line demonstrated that cell proliferation was enhanced, but cell invasion was suppressed by conditioned medium [13]. HCC development is associated with multiple signalling pathways [22–25]. Zhao et al. (2012) concluded that the Akt signalling pathway, which regulates tumour cell growth and survival, was downregulated by AT-MSC-CM. Conversely, Li et al. (2010) suggested that the TGFβ1 pathway, which is associated with tumour cell progression and generating a favourable microenvironment for tumour cell growth, was suppressed by AT-MSC-CM. However, in our study using a canine HCC cell line, the TGFβ1 expression level was increased by culture with AT-MSC-CM. A previous study suggested that MSCs enhanced tumour growth but inhibited the invasiveness and metastasis of HCC by downregulating TGFβ1 [13]. TGFβ mRNA and protein levels were reduced by feedback mechanisms due to abundant TGFβ in AT-MSC-CM. TGFβ is an oncogenic factor with roles in proliferation, angiogenesis, invasion, and metastasis during tumour progression [26,27]. Moreover, TGFβ closely interacts with cellular and non-cellular components of the tumour microenvironment to promote tumour progression. TGFβ is regulated by positive and negative feedback mechanisms through various pathways [28]. Moreover, an autocrine effect of TGFβ has been demonstrated in tumour cells [29]. In mammary carcinoma patients, TGFβ secreted by tumours contributed to the formation of a favourable microenvironment for tumour growth and spread by acting directly on the tumour cells [30]. Therefore, we suggest that TGFβ in the AT-MSC-CM may have induced the increase in TGFβ expression levels observed in AZACH cells cultured with AT-MSC-CM in our study.

In addition to TGFβ1, mRNA expression levels of the growth factors EGF, HGF, PDGFβ, VEGFA, and IGF2 were significantly increased in canine HCC cells cultured with AT-MSC-CM. HGF mRNA levels, in particular, were markedly increased after culture with AT-MSC-CM. cMet is an HGF receptor; HGF–cMet axis activation is implicated in cellular invasion and metastasis through promotion of proliferation, migration, mobility, three-dimensional epithelial cell organization, and angiogenesis in HCC [25,31]. The other growth factors whose mRNA expression levels increased in cells cultured with AT-MSC-CM in this study also promote HCC progression by enhancing processes such as development, proliferation, metastasis, and angiogenesis [32]. For instance, EGF plays an important role in tumour proliferation and metastasis [33]; PDGFβ induces liver fibrosis and accelerates tumour development [34,35]; VEGFA is one of the major growth factors responsible for angiogenesis [36]; and IGF2 enhances tumour cell proliferation [37]. We did not examine in detail how the expression levels of these growth factors might be related to tumour progression. However, the changes might be related to the observed upregulation of canine HCC cell proliferation and invasion, given that increased mRNA expression levels of these growth factors have been demonstrated to be crucial to tumour progression [25].

A critical event in tumour cell invasion is degradation of the extracellular matrix (ECM), which acts as a barrier to cancer cell spread to distal sites by restricting tumour growth and invasion [38]. Among the many known MMPs, gelatinases, especially MMP2 and MMP9, are thought to play key roles in degrading type IV collagen and gelatin, the two main components of the ECM. MMP2 and MMP9 expression is associated with tumour cell invasion, and is elevated in various malignancies including HCC [38–40]. In our study, MMP2 mRNA expression level was significantly increased in cells cultured with AT-MSC-CM, whereas MMP9 mRNA expression level was decreased. MMP2 and MMP9 expression are highly regulated by cytokines and complex signalling pathways, and thus may be controlled by distinct mechanisms in the responses to canine AT-MSC-CM observed in this study. Studies using human HCC cell lines demonstrated that HCC cells with higher MMP2 expression exhibited higher viability and migration ability [41,42]. Conversely, another study found that expression of MMP9, not MMP2, was essential for HCC invasion and progression [39]. However, high expression of MMP2 and MMP9, alone and in combination has been associated with tumour progression in HCC. Therefore, the increased MMP2 mRNA expression level that we observed in AZACH cells cultured with AT-MSC-CM might be associated with the increase in proliferation and invasion of these cells. However, our study has focused on clarifying the expression of mRNAs, and further studies are needed to evaluate the protein expression levels and mechanistic pathways of these genes.

The AT-MSC secretome is rich in proteins including cytokines, chemokines, and growth factors [20,21], and thus has the potential to be a useful tool for treatment of various diseases. Numerous studies have considered the beneficial effects exerted by the AT-MSC secretome on processes including angiogenesis, immunomodulation, wound healing, and tissue regeneration [43]. However, the effects of soluble factors from AT-MSCs on tumour cells are not well known, and the published results are controversial. Many reports have indicated that AT-MSCs promote the growth, progression, and metastatic spread of human breast cancer tumour cells [44]. Moreover, research using a human pancreatic cell line demonstrated that AT-MSCs promoted pancreatic cancer cell proliferation and invasion [45].

Our study has several important limitations. First, the effects of soluble factors from AT-MSC-CM was examined using only one canine HCC cell line, because there were no other canine HCC cell lines commercially available. Therefore further studies are needed using other cell lines. Second, our study demonstrated that the ability of HCC cell invasion was upregulated when cultured with AT-MSC-CM. However, this alteration induced by whether the direct influence of soluble factors from canine AT-MSCs or results in upregulation of HCC cells proliferation is still unclear. Therefore, proteomic analyses are needed in canine AT-MSC-CM to confirm whether soluble factors from AT-MSC are including proteins effect on HCC cells invasiveness. Thus, future studies should address how soluble factors from AT-MSCs influence tumour cell growth and metastasis.

## Conclusion

Our results show that soluble factors from canine AT-MSCs promote HCC proliferation and invasion. Our findings indicate that upregulation of growth factors and MMP2 may contribute to these changes.
